# Multimodal prediction of major adverse cardiovascular events in hypertensive patients with coronary artery disease: integrating pericoronary fat radiomics, CT-FFR, and clinicoradiological features

**DOI:** 10.1007/s11547-025-01991-3

**Published:** 2025-03-21

**Authors:** Qing Zou, Taichun Qiu, Chunxiao Liang, Fang Wang, Yongji Zheng, Jie Li, Xingchen Li, Yudan Li, Zhongyan Lu, Bing Ming

**Affiliations:** 1 Department of Radiology, Deyang People’s Hospital, 173# Section 3 Tai Shan Road, Deyang, 618400 Sichuan China; 2 Department of Research and Development, Shanghai United Imaging Intelligence Co., Ltd., Shanghai, 200232 China

**Keywords:** Pericoronary adipose tissue, Major adverse cardiovascular events, Radiomics, Coronary artery disease, Hypertension

## Abstract

**Purpose:**

People with both hypertension and coronary artery disease (CAD) are at a significantly increased risk of major adverse cardiovascular events (MACEs). This study aimed to develop and validate a combination model that integrates radiomics features of pericoronary adipose tissue (PCAT), CT-derived fractional flow reserve (CT-FFR), and clinicoradiological features, which improves MACE prediction within two years.

**Materials and methods:**

Coronary-computed tomography angiography data were gathered from 237 patients diagnosed with hypertension and CAD. These patients were randomly categorized into training and testing cohorts at a 7:3 ratio (165:72). The least absolute shrinkage and selection operator logistic regression and linear discriminant analysis method were used to select optimal radiomics characteristics. The predictive performance of the combination model was assessed through receiver operating characteristic curve analysis and validated via calibration, decision, and clinical impact curves.

**Results:**

The results reveal that the combination model (Radiomics.Clinical.Imaging) improves the discriminatory ability for predicting MACE. Its predictive efficacy is comparable to that of the Radiomics.Imaging model in both the training (0.886 vs. 0.872) and testing cohorts (0.786 vs. 0.815), but the combination model exhibits significantly improved specificity, accuracy, and precision. Decision and clinical impact curves further confirm the use of the combination prediction model in clinical practice.

**Conclusions:**

The combination prediction model, which incorporates clinicoradiological features, CT-FFR, and radiomics features of PCAT, is a potential biomarker for predicting MACE in people with hypertension and CAD.

**Supplementary Information:**

The online version contains supplementary material available at 10.1007/s11547-025-01991-3.

## Introduction

Cardiovascular disease remains the leading cause of global mortality, with coronary artery disease (CAD) accounting for > 50% of all major adverse cardiovascular events (MACE) in adults aged < 75 years. According to the 2022 China Health Report, hypertension is an independent risk factor for CAD development [1]. Notably, hypertension and CAD frequently coexist, with their pathophysiological interplay characterized by complex mechanisms such as accelerated atherosclerotic plaque formation, vascular damage, and the interplay between arterial stiffness and coronary perfusion. Patients with coexisting hypertension and CAD face a significantly heightened risk of cardiovascular mortality [2–4]. Thus, the development of accurate and effective tools for early identification of individualized MACE risk, alongside timely intervention and improved prognostic management strategies, is crucial to alleviating the burden in this high-risk population with coexisting hypertension and CAD.

The 2019 European Society of Cardiology (ESC) guidelines recommended coronary CT angiography (CCTA) as the first-tier noninvasive imaging modality for the evaluation of suspected CAD [5]. Traditionally, CCTA has relied on the detection of obstructive lesions or coronary calcification to guide cardiovascular risk stratification and inform clinical decision-making. However, even with optimal pharmacological therapy and comprehensive clinical risk management, many patients experience residual cardiovascular risk, leading to persistent MACE occurrence. Moreover, the positive predictive value of CCTA is often compromised by the limitations of CT spatial resolution and challenges posed by severe coronary calcification, resulting in uncertainties in risk stratification and healthcare intervention targeting.

In recent years, CT-derived fractional flow reserve (CT-FFR) and perivascular adipose tissue (PCAT) analysis, both derived from CCTA images, have demonstrated significant value in the functional assessment of CAD and the detection of inflammatory states. CT-FFR integrates anatomical and functional assessments, substantially improving the diagnostic accuracy for myocardial ischemia and informing clinical decision-making [6]. Meanwhile, PCAT, as a marker of coronary artery inflammation, has been shown to correlate closely with cardiovascular mortality and the need for revascularization [7, 8]. Furthermore, studies have revealed that prediction models developed using PCAT-based radiomics outperform traditional indicators in forecasting MACE [9]. Despite these advances, significant gaps remain in the literature regarding the application of PCAT radiomics in high-risk patients with coexisting hypertension and CAD. To address this gap, this prospective case–control study will leverage CCTA-based radiomic analysis of PCAT, in conjunction with CT-FFR and clinicoradiological features, to evaluate its utility in predicting MACE. The study aims to develop and validate a multimodal predictive model for MACE risk over a two-year follow-up period, potentially advancing personalized risk stratification and management in this high-risk population.

## Materials and methods

### Patients

**Fig. 1:**
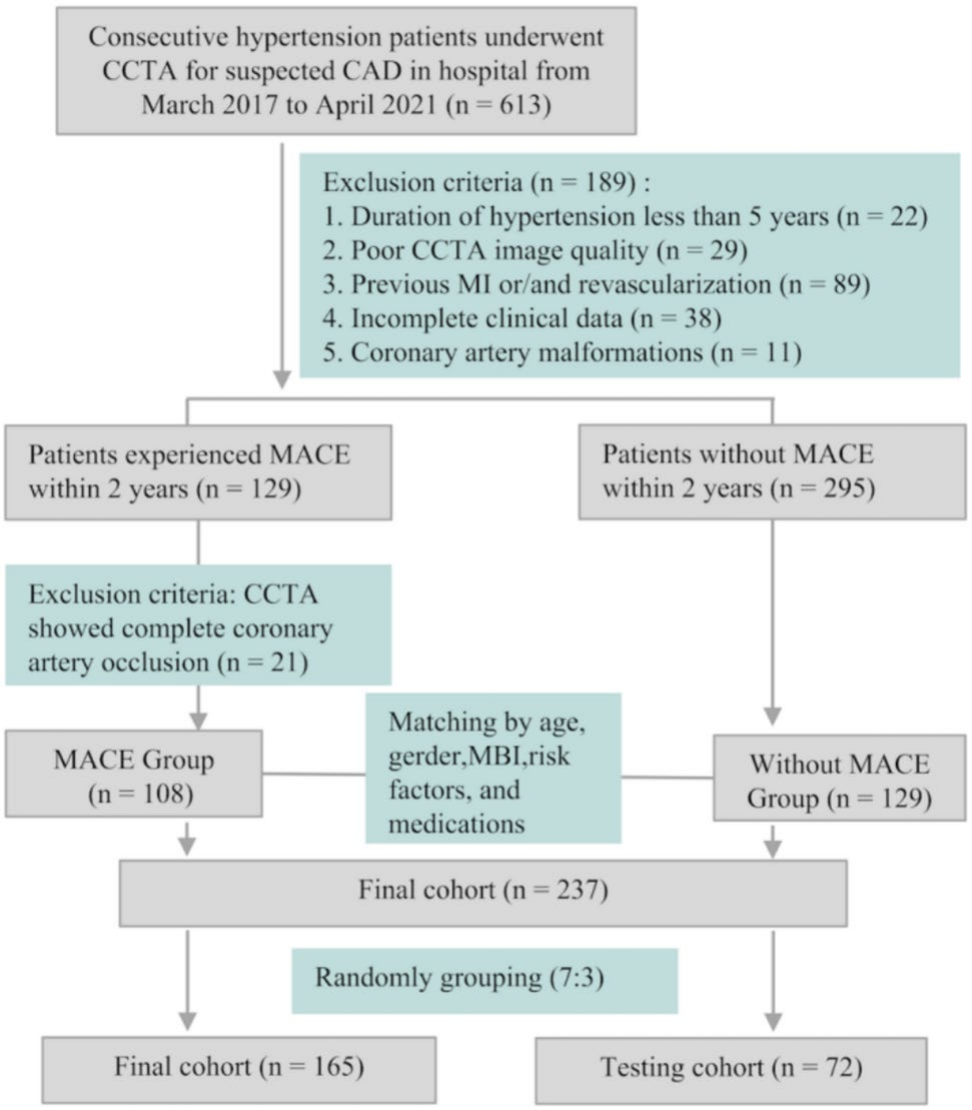
Study flowchart of the enrolled patients

The study (ethical code: 2023-04-097-K01) was approved by our hospital ethics review committee. Informed consent was waived because CCTA image data acquisition is part of the routine examination protocol for patients with CAD. The study retrospectively included patients with hypertension who underwent a CCTA scan from January 2017 to April 2021 due to suspected CAD. According to international guidelines, hypertension was defined based on repeated office systolic blood pressure (SBP) values 140 mmHg and/or diastolic blood pressure (DBP) 90 mmHg [10]. Blood pressure (BP) measurements were conducted by trained healthcare professionals, including nurses, physicians, and technicians. During each measurement, two to three BP readings were taken at intervals of 1–2 min. All BP assessments adhered to standardized protocols: patients rested in a seated position for at least 5 min before measurement. Validated upper-arm automated blood pressure monitors were used, and the procedure was conducted in strict accordance with established guidelines to ensure reliability and reproducibility. All patients who experienced MACE within 2 years after the last CCTA examination were analyzed. The study endpoint was defined as composite MACE including cardiac death, myocardial infarction, hospitalization for acute heart failure, and revascularization. Exclusion criteria included patients with a hypertension duration of < 5 years (*n* = 22), to account for the impact of hypertension progression on CAD and ensure the stability of results [11, 12]; poor CCTA image quality unsuitable for analysis (*n* = 29); previous myocardial infarction and/or revascularization (*n* = 89); incomplete clinical data (*n* = 38); coronary artery malformations (*n* = 11); and complete coronary artery occlusion, precluding CT-FFR and perivascular fat attenuation index (FAI) analysis (*n* = 21). Ultimately, this study included 108 and 129 patients with and without MACE, with the latter matched by age, gender, risk factors, and medication. Figure 1 shows the patient selection flowchart. We randomly categorized 237 patients into the training (*n* = 165, 76 in the MACE group and 89 in the without MACE group) and testing cohorts (*n* = 72, 32 in the MACE group and 40 in the without MACE group) at a 7:3 ratio.

### Clinical features retrieval and follow-up

The demographic data were collected for all patients, including age, sex, height/weight, systolic/diastolic blood pressure, cardiac function grade (New York Heart Association, NYHA), hypertension duration (years), comorbidities, medication, as well as cardiovascular and cerebrovascular history*.* Table 1 shows the laboratory data, including measurements of white blood cell count, C reactive protein, creatine kinase MB (CKMB), troponin, blood glucose concentration, lipid profile, and thyroid function tests. A cardiologist, blinded to the CCTA findings, was responsible for reviewing patient charts, clinical presentations, and identifying composite MACE. Data were systematically collected through electronic medical records from participating centers and standardized telephone interviews using structured follow-up questionnaires. The timing of each event was meticulously recorded. Participants were followed up for 2 years after discharge. For those who survived without MACE, the 2-year follow-up administratively concluded by April 2023. Mortality unrelated to MACE was recorded separately, and follow-up was terminated upon such events.

**Table 1:** Comparison of characteristics between training and testing patients with and without MACE

Characteristics	Training cohort			Testing cohort			*p*_Train vs. Test_
	MACE− (*n* = 89)	MACE+ (*n* = 76)	*p*	MACE− (*n* = 40)	MACE+ (*n* = 32)	*p*	
*Demographic characteristics*							
Age (Median [Q1 ~ Q3])	73 [67 ~ 79]	73 [67.75 ~ 80]	0.932	72 [68 ~ 77]	71 [68 ~ 77]	0.849	0.314
Gender (%)			0.995			0.361	0.785
Female	34 (38.2)	29 (38.2)		18 (45.0)	11 (34.4)		
Male	55 (61.8)	47 (61.8)		22 (55.0)	21 (65.6)		
Height (Median [Q1 ~ Q3])	160 [156 ~ 165]	160 [155 ~ 167.25]	0.916	159 [155 ~ 165]	160 [157 ~ 165]	0.344	0.792
Weight (mean ± sd)	60 [55 ~ 66]	61.5 [54 ~ 70]	0.823	60 [53 ~ 67]	60 [55 ~ 65.5]	0.878	0.563
SBP (Median [Q1 ~ Q3])	138 [125 ~ 162]	138.5 [121 ~ 163.25]	0.678	141.5 [132.2 ~ 160]	155 [136 ~ 171]	0.209	0.037
DBP (mean ± sd)	80.539 ± 14.686	79.289 ± 15.172	0.593	81.275 ± 12.983	85.312 ± 16.263	0.258	0.207
Hypertension history (Median [Q1 ~ Q3])	10 [5 ~ 17]	10 [6.5 ~ 20]	0.292	10 [5 ~ 10]	10 [6.5 ~ 10]	0.760	0.238
Diabetes history (Median [Q1 ~ Q3])	0 [0 ~ 6]	1 [0 ~ 10]	0.032	0 [0 ~ 4]	0 [0 ~ 4.25]	0.753	0.077
NYHA grade (%)			0.030			0.950	0.371
I	21 (23.6)	18 (23.7)		12 (30.0)	6 (18.8)		
II	41 (46.1)	23 (30.3)		19 (47.5)	13 (40.6)		
III	21 (23.6)	19 (25.0)		9 (22.5)	9 (28.1)		
IV	6 (6.7)	16 (21.1)		0 (0.0)	4 (12.5)		
COPD (%)			0.812			1.000	0.859
Negative	75 (84.3)	63 (82.9)		34 (85.0)	27 (84.4)		
Positive	14 (15.7)	13 (17.1)		6 (15.0)	5 (15.6)		
Hyperlipidemia (%)			0.274			0.024	0.866
Negative	74 (83.1)	58 (76.3)		36 (90.0)	22 (68.8)		
Positive	15 (16.9)	18 (23.7)		4 (10.0)	10 (31.2)		
Chronic kidney diseases (%)			0.076			0.720	0.883
Negative	81 (91.0)	62 (81.6)		34 (85.0)	29 (90.6)		
Positive	8 (9.0)	14 (18.4)		6 (15.0)	3 (9.4)		
Diabetes (%)			0.041			0.402	0.071
Negative	54 (60.7)	34 (44.7)		25 (62.5)	23 (71.9)		
Positive	35 (39.3)	42 (55.3)		15 (37.5)	9 (28.1)		
Antihypertensive treatment (%)			0.362			0.002	0.507
Negative	19 (21.3)	12 (15.8)		15 (37.5)	2 (6.2)		
Positive	70 (78.7)	64 (84.2)		25 (62.5)	30 (93.8)		
Statin use (%)			0.087			0.262	0.328
Negative	47 (52.8)	30 (39.5)		19 (47.5)	11 (34.4)		
Positive	42 (47.2)	46 (60.5)		21 (52.5)	21 (65.6)		
Smoking (%)			0.341			0.172	0.897
Negative	69 (77.5)	54 (71.1)		33 (82.5)	22 (68.8)		
Positive	20 (22.5)	22 (28.9)		7 (17.5)	10 (31.2)		
Drinking (%)			0.278			0.687	0.610
Negative	76 (85.4)	60 (78.9)		35 (87.5)	26 (81.2)		
Positive	13 (14.6)	16 (21.1)		5 (12.5)	6 (18.8)		
DLP (Median [Q1 ~ Q3])	170 [130 ~ 238]	206 [146.25 ~ 259]	0.086	175 [128.475 ~ 221.5]	200.5 [165.5 ~ 216.425]	0.117	0.959
*Laboratory characteristics*							
FT3 (Median [Q1 ~ Q3])	4.245 [3.828 ~ 4.848]	3.935 [3.575 ~ 4.502]	**0.020**	4.350 [3.820 ~ 4.785]	3.980 [3.570 ~ 4.505]	**0.021**	0.762
FT4 (Median [Q1 ~ Q3])	16.37 [14.19 ~ 17.773]	16.1 [14.3 ~ 18.517]	0.754	15.815[14.203 ~ 17.27]	16.11 [14.91 ~ 18.25]	0.348	0.483
TSH (Median [Q1 ~ Q3])	3.13 [2.02 ~ 4.452]	2.66 [1.832 ~ 4.035]	0.228	2.725 [1.94 ~ 5.025]	2.88 [1.31 ~ 4.54]	0.800	0.993
TGAb (Median [Q1 ~ Q3])	13.02 [10 ~ 17.465]	11.6 [10 ~ 15.367]	0.142	14.325 [12.875 ~ 17.445]	11.96 [10 ~ 16.26]	0.095	0.110
TPOAb (Median [Q1 ~ Q3])	12.62 [8.8 ~ 19.225]	11.505 [7.908 ~ 16.913]	0.263	10.7 [8.55 ~ 18.122]	14.35 [10.51 ~ 25.01]	0.208	0.660
C reactive protein (Median [Q1 ~ Q3])	0.61 [0.499 ~ 2.44]	4.58 [0.5 ~ 17.41]	0.002	1.335 [0.5 ~ 19.04]	1.18 [0.499 ~ 4.34]	0.293	0.868
White blood cell count (Median [Q1 ~ Q3])	6 [5.12 ~ 7.64]	6.63 [5.48 ~ 8.36]	0.165	6.505 [5.318 ~ 8.137]	6.24 [5.73 ~ 8.43]	0.835	0.227
Creatinine (Median [Q1 ~ Q3])	76.05 [65.4 ~ 90.125]	81.4 [67.4 ~ 104]	0.175	77.6 [69.6 ~ 100.6]	79 [73.2 ~ 93.8]	0.590	0.580
Troponin (Median [Q1 ~ Q3])	0.009 [0.002 ~ 0.023]	0.054 [0.008 ~ 0.392]	** < 0.01**	0.011 [0.001 ~ 0.036]	0.057 [0.008 ~ 0.713]	**0.003**	0.553
CKMB (Median [Q1 ~ Q3])	1.24[0.898 ~ 1.812]	1.99 [1.195 ~ 3.05]	< 0.01	1.32 [0.85 ~ 2.29]	2.33 [1.365 ~ 3.66]	0.064	0.363
MYO (Median [Q1 ~ Q3])	44.42[36.943 ~ 62.565]	61.78 [39.97 ~ 105.77]	0.021	57.26 [45.69 ~ 77.44]	53.17 [34.52 ~ 80.51]	0.299	0.544
*Imaging characteristics*							
**LAD DS (%)**			0.058			0.987	0.751
No (0%)	12 (13.5)	5 (6.6)		2 (5.0)	2 (6.2)		
Minimal (1–24%)	15 (16.9)	10 (13.2)		13 (32.5)	2 (6.2)		
Mild (25–49%)	22 (24.7)	13 (17.1)		6 (15.0)	4 (12.5)		
Moderate (50–69%)	20 (22.5)	15 (19.7)		10 (25.0)	6 (18.8)		
Severe (≥ 70%)	20 (22.5)	33 (43.4)		9 (22.5)	18 (56.2)		
**LCX DS (%)**			0.001			0.994	0.952
No (0%)	42 (47.2)	17 (22.4)		21 (52.5)	7 (21.9)		
Minimal (1–24%)	19 (21.3)	16 (21.1)		10 (25.0)	5 (15.6)		
Mild (25–49%)	14 (15.7)	13 (17.1)		2 (5.0)	10 (31.2)		
Moderate (50–69%)	9 (10.1)	11 (14.5)		4 (10.0)	5 (15.6)		
Severe (≥ 70%)	5 (5.6)	19 (25.0)		3 (7.5)	5 (15.6)		
**RCA DS (%)**			0.001			0.996	0.434
No (0%)	24 (27.0)	16 (21.1)		10 (25.0)	1 (3.1)		
Minimal (1–24%)	25 (28.1)	15 (19.7)		16 (40.0)	7 (21.9)		
Mild (25–49%)	21 (23.6)	6 (7.9)		3 (7.5)	9 (28.1)		
Moderate (50–69%)	11 (12.4)	20 (26.3)		7 (17.5)	8 (25.0)		
Severe (≥ 70%)	8 (9.0)	19 (25.0)		4 (10.0)	7 (21.9)		
**DS Patient level (%)**			0.001			0.999	0.343
*CAD-RADS* 0	7 (7.9)	2 (2.6)		0 (0.0)	1 (3.1)		
*CAD-RADS* 1	14 (15.7)	7 (9.2)		12 (30.0)	2 (6.2)		
*CAD-RADS* 2	18 (20.2)	11 (14.5)		6 (15.0)	2 (6.2)		
*CAD-RADS* 3	26 (29.2)	12 (15.8)		13 (32.5)	5 (15.6)		
*CAD-RADS* 4	24 (27.0)	44 (57.9)		9 (22.5)	22 (68.8)		
Positive remodeling (%)			0.755			0.673	0.311
*Negative*	49 (55.1)	40 (52.6)		18 (45.0)	16 (50.0)		
*Positive*	40 (44.9)	36 (47.4)		22 (55.0)	16 (50.0)		
Punctate calcification (%)			0.076			0.697	0.173
*Negative*	81 (91.0)	62 (81.6)		31 (77.5)	26 (81.2)		
*Positive*	8 (9.0)	14 (18.4)		9 (22.5)	6 (18.8)		
Napkin Ring (%)			0.559			0.026	0.185
*Negative*	84 (94.4)	70 (92.1)		39 (97.5)	25 (78.1)		
*Positive*	5 (5.6)	6 (7.9)		1 (2.5)	7 (21.9)		
Low density spots (%)			0.327			0.402	0.485
*Negative*	67 (75.3)	52 (68.4)		25 (62.5)	23 (71.9)		
*Positive*	22 (24.7)	24 (31.6)		15 (37.5)	9 (28.1)		
HRP (%)			0.043			0.624	0.219
*Negative*	69 (77.5)	48 (63.2)		24 (60.0)	21 (65.6)		
*Positive*	20 (22.5)	28 (36.8)		16 (40.0)	11 (34.4)		
Calcification score (Median [Q1 ~ Q3])	195.18 [16.6 ~ 962.81]	353.145 [102.525 ~ 827.955]	0.148	220.76 [111.898 ~ 446.413]	455.83 [148.488 ~ 615.062]	0.114	0.997
Involved segments (Median [Q1 ~ Q3])	4 [2 ~ 6]	6 [3 ~ 8]	**0.008**	4 [3 ~ 5]	6 [3.75 ~ 8]	**0.013**	0.805
LAD CT-FFR (Median [Q1 ~ Q3])	0.89 [0.79 ~ 0.94]	0.745 [0.65 ~ 0.88]	**< 0.01**	0.875 [0.777 ~ 0.932]	0.74 [0.675 ~ 0.835]	**0.001**	0.441
LCX CT-FFR (Median [Q1 ~ Q3])	0.93 [0.84 ~ 0.96]	0.86[0.718 ~ 0.923]	**< 0.01**	0.915 [0.88 ~ 0.952]	0.895 [0.8 ~ 0.932]	**0.049**	0.873
RCA CT-FFR (Median [Q1 ~ Q3])	0.92 [0.83 ~ 0.96]	0.85 [0.688 ~ 0.91]	**< 0.01**	0.92 [0.862 ~ 0.96]	0.85 [0.732 ~ 0.92]	**0.005**	0.419
LAD FAI (mean ± sd)	− 88.966 ± 9.071	− 86.5 ± 9.87	0.099	− 90 ± 10.031	− 86.69 ± 9.650	0.107	0.581
LCX FAI (mean ± sd)	− 83.202 ± 8.367	− 80.039 ± 8.951	0.021	− 82.925 ± 8.827	− 79.281 ± 8.141	0.074	0.705
RCA FAI (Median[Q1 ~ Q3])	− 92 [− 100 ~ -84]	− 88 [− 97 ~ − 81]	0.059	− 90.6 ± 11.41	− 87.938 ± 11.865	0.339	0.708
LAD Fat volume (Median [Q1 ~ Q3])	1676 [1376 ~ 2006]	1655 [1258.25 ~ 1987]	0.635	1720 [1404 ~ 2045]	1640 [1237 ~ 2001]	0.357	0.646
LCX Fat volume (mean ± sd)	1241.079 ± 461.291	1070.066 ± 488.308	0.023	1207.2 ± 462.543	1065 ± 473.043	0.205	0.831
RCA Fat volume (Median [Q1 ~ Q3])	2173.5 [1810.25 ~ 2476.5]	2115 [1576.25 ~ 2363.75]	0.172	2279 [1834.75 ~ 2611.25]	2224.5 [1883.75 ~ 2410]	0.344	0.256

Bold values highlight the P-values with statistical differences

Values are mean ± standard deviation, median [25th and 75th percentile] or n (%)

SBP, systolic blood pressure; DBP, diastolic blood pressure; NYHA, New York Heart Association; COPD, chronic obstructive pulmonary disease; DLP, dose-length product; FT3, free triiodothyronine; FT4, free thyroxine; TSH, thyroid-stimulating hormone; TGAb, thyroglobulin antibody; TPOAb, thyroid peroxidase antibody; CKMB, creatine kinase MB; MYO, myoglobin; DS, diameter stenosis; CAD-RADS, Coronary Artery Disease-Reporting and Data System; HRP, high-risk plaques; LAD, left anterior descending artery; LCX, left circumflex artery; RCA, right coronary artery; HRP, high-risk plaques; CT-FFR, computed tomography-based fractional flow reserve; FAI, perivascular fat attenuation index

### CT image acquisition protocol

The CCTA images were acquired using dual-source CT scanners (SOMATOM Force, Siemens Healthineers, Germany) following the Society of Cardiovascular Computed Tomography guidelines [13]. Patients were trained to breathe before the examination. The CCTA protocols for image acquisition were as follows: Collimator of 192 × 0.6 mm, pitch of 3.2, 0.25 s/rot, tube voltage of 70–100 kVp, tube current automatically adjusted, layer thickness of 0.75 mm, and rotation time of 280 mAs. The acquisition time window was controlled at 30–80% of the R-R interval. Initially, a calcium score was performed. A contrast agent (ultravist of 370; Bayer, Germany; 1.5 ml/kg) was then injected through the anterior elbow vein at a flow rate of 4.5–5.0 ml/s using a high-pressure injector, followed by a 40 ml flush of saline at the same rate. The bolus tracking method with a region of interest placed at the ascending aorta and the attenuation threshold set as 100 Hounsfield units were used for image acquisition. Tube current was automatically adjusted according to the patient’s body habitus by an automatic exposure control system (CARE Dose, Siemens Healthineers) and electrocardiographically modified.

### Coronary plaque analysis

All post-processing analyses of CCTA images were performed at end-diastole. Two experienced radiologists independently assessed imaging features, with any discrepancies were resolved through consensus. Axial and multiplanar reconstruction were performed on an offline workstation (Syngo. Via, version VB20, Siemens Healthineers) to analyze CCTA images. The coronary segments were visually examined for the presence of plaques, which were characterized as calcified or non-calcified. The quantitative and qualitative analysis of coronary plaques adhered to Coronary Artery Disease -Reporting and Data System [14]. Coronary diameter stenoses (DS) of left anterior descending artery (LAD), right coronary artery (RCA), and left circumflex artery (LCX) were classified as no visible stenosis (0%), minimal stenosis (1–24%), mild stenosis (25–49%), and moderate stenosis (50–69%). Stenosis of 70–99%, left main stenosis > 50%, or 3-vessel obstructive disease (≥ 70%) were all classified as severe stenosis. The highest-grade stenotic lesions in each main vessel and patient (DS _patient_) were recorded. Plaque burden evaluation included the coronary calcification score and the number of segments involved with plaque using the 18-segment coronary model. The coronary artery calcium (CAC) testing (Agatston method) was used to identify the total amount of calcified plaque. The segment involvement score (SIS) was calculated by summing the number of coronary artery segments where plaque is present, irrespective of severity. Both CAC and SIS were utilized to ensure that non-calcified plaque is also accounted [15].

High-risk plaques (HRPs) were classified as plaques exhibiting two or more of vulnerable features simultaneously as follows: (1) low-attenuation plaque: plaque with an average density ≤ 30 HU; (2) positive remodeling: a lesion-to-reference diameter ratio ≥ 1.1; (3) napkin-ring sign: A ring-like attenuation pattern with peripheral high-attenuation tissue surrounding a central lower-attenuation area; 4) spotty calcification: plaque with an average density > 130 HU and a diameter < 3 mm [16].

### CT-FFR analysis

We used the coronary CT-FFR semiautomatic quantification software (version 1.11.1, Shukun [Beijing] Network Technology) for analysis. To ensure comprehensive evaluation, CT-FFR of LAD, RCA, and LCX was assessed. The measurement approach varied based on the presence of plaque. For vessels with plaque, the lesion-based method was employed, with CT-FFR measured 20-mm distal-to-the-most-severe lesion [17, 18]. For vessels without plaque or stenosis, the diameter-based method was used, measuring CT-FFR at the distal segment of vessels with a diameter of 2 mm [19].

### PCAT segmentation

PCAT was quantitatively measured using the uAI Research Portal (version 20230915, Shanghai United Imaging Intelligence Co., Ltd) [20]. It is defined as adipose tissue located within a radial distance from the outer vessel wall equal to the diameter of the coronary artery and with a CT attenuation range of − 190 HU to − 30 HU [21]; the mean CT value of pericoronary fat within this range is referred to as the fat attenuation index (FAI). Automated tracking was used to measure adipose tissue around the three coronary trunks, spanning a 40-mm length range proximal to the LAD and LCX, and a 10–50 mm length segment proximal to the RCA [21]. The FAI for the LAD, RCA, and LCX was calculated; the fat volume was also recorded.

### PCAT radiomics feature extraction

The Pyradiomics package (version 3.0.1) was employed to resample the CCTA images with a voxel size of 1 mm × 1 mm × 1 mm, utilizing a linear interpolation algorithm to standardize the voxel spacing. Voxel intensity values were discretized with a fixed bin width of 25 Hounsfield units to control for image noise. Radiomics feature extraction was performed using the uAI research portal. A total of 1904 radiomics features were extracted from each PCAT, including 450 first-order, 14 shape, and 1512 texture features. Among them, 104 and 1800 features were derived from the original and filtered processed images, respectively. Table S1 shows more radiomics feature information**.**

### Feature selection and model construction

The process of selecting features is conducted on the training cohort. Different feature selection methods are used for radiomics and clinicoradiological features. Prior to analysis, z-score normalization was employed to eliminate differences in indicator dimensionality for the radiomics features. The study used Spearman rank correlation coefficient, maximum correlation and minimum redundancy, and least absolute shrinkage and selection operator (LASSO) methods to select the most suitable MACE-predicted features. Univariate and multivariate logistic regression analyses were used to assess the independent predictors of MACE based on clinicoradiological features. Predictive features were identified using the change in estimate (CIE) method based on univariate logistic regression analyses if no independent predictors were found in the multivariate logistic regression analyses (*p* > 0.05).

**Fig. 2:**
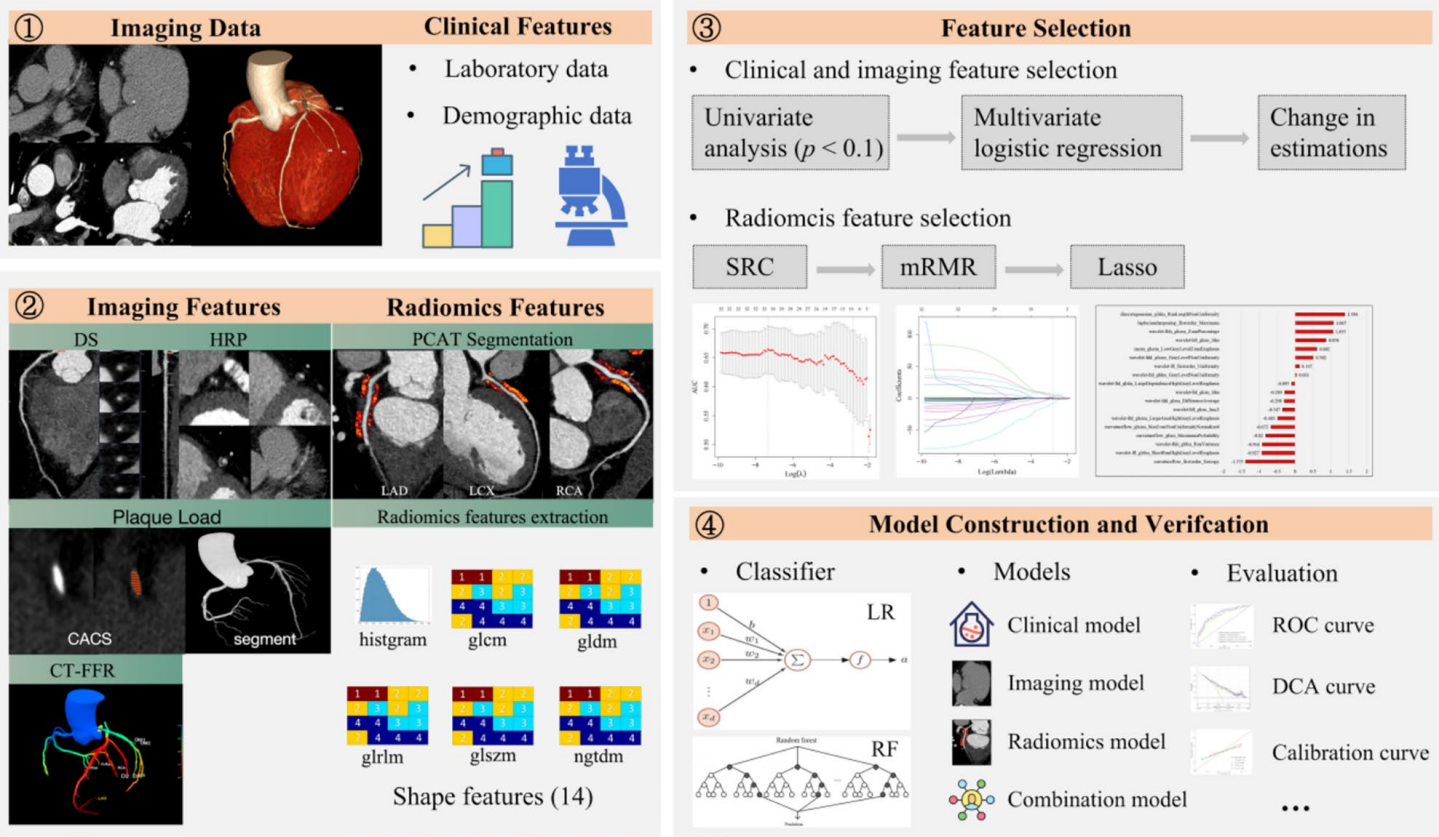
Study flowcharts of radiomics analysis

Logistic regression (LR), random forest (RF), and support vector machine (SVM) were used to develop the MACE predictive models. Initially, we developed five individual models: a clinical model, a radiological (imaging) model, and the LAD, LCX, and RCA PCAT radiomics model. Subsequently, we combined radiomics features from the LAD, LCX, and RCA to identify the final MACE radiomics model. Finally, we integrated the radiomics and clinical-radiological features to construct the integrated models. The objective of linear discriminant analysis (LDA) is to maximize the inter-class variance and minimize the intra-class variance. This is accomplished by projecting a high-dimensional feature space into a low-dimensional space, which enhances the efficacy of subsequent predictive models. Consequently, after identifying the optimal predictive features of each model, this study conducted LDA analysis and subsequently constructed LR, RF, and SVM models. Receiver operating characteristics curves were used to assess model performance. The area under the curve (AUC), sensitivity, specificity, accuracy, precision, and F1-score were calculated. The Delong test was used to compare prediction efficiency, and the Benjamini–Hochberg method was utilized to correct the false discovery rate. The Hosmer–Lemeshow test was used to evaluate the agreement between the actual and the predicted incidence rates of MACE. Calibration curves were created, and decision curves were used to validate the clinical usefulness of the model by measuring the net benefit under different risk thresholds. Figure 2 illustrates the radiomics analysis workflow.

### Correlation analysis

Radiomics features extracted from PCAT were correlated with MACE, CKMB, DS_patient_, HRP, LAD FFR, LCX DS, LCX FAI, RCA FFR, and troponin, respectively, and heatmap was performed. Biserial correlation analysis was used to calculate the correlation coefficients of continuous and binary variables, while Pearson or Spearman correlation analysis was used for continuous and continuous variables. This approach was selected due to the differing characteristic ranges and distributions.

### Statistical analysis

Student’s *t*-test was used for normally distributed variables, while the Mann–Whitney U test was used for non-normally distributed continuous variables, and the Chi-square test for qualitative variables to analyze statistically significant differences. Statistical analysis was performed using R software (version 4.1.3), with all tests being two-sided and *p*-values of < 0.05 considered statistically significant.

## Result

### Performance of baseline model

The study included 237 patients, with 165 and 72 cases assigned to the training and testing cohorts, respectively. Among them, 76 MACE cases were determined in the training cohort and 32 in the testing cohort. Table 1 shows the clinical features, laboratory data, and imaging features of all patients included. Both the training and testing cohorts demonstrated significant differences between MACE group (MACE+) and without MACE group (MACE-) in FT3, troponin, involved segments, LAD FFR, LCX FFR, and RCA FFR. Univariate analysis revealed 15 clinicoradiological features to be significantly associated with MACE, but a multivariate analysis indicated no independent predictors (*p* > 0.05) (Table S2). Ultimately, the final input features for the clinical model after controlling the confounders by CIE were CKMB and troponin, while the imaging model included HRP, LAD FFR, RCA FFR, LCX FAI, LCX DS, and DS_patient_.

**Fig. 3:**
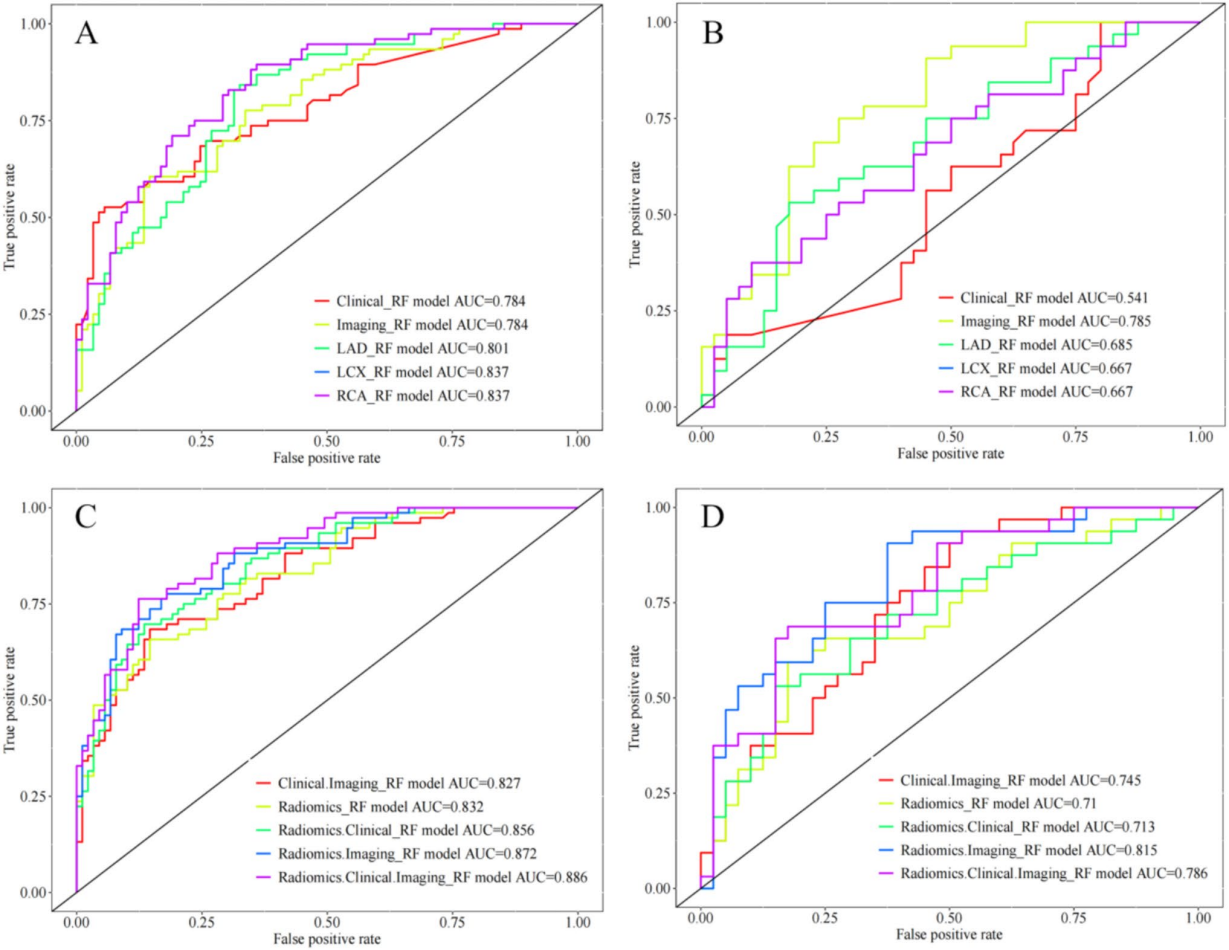
Comparison of receiver operating characteristics (ROC) curves for MACE prediction in training (A,C),and testing (B,D) cohorts by random forest

The Imaging model demonstrated superior discrimination compared to the clinical model, with an AUC of 0.784 in the training cohort and 0.785 in the testing cohort, versus 0.784 and 0.541, respectively. The radiomics models established by LAD, LCX, and RCA demonstrated that the LCXR in the training group had a higher AUC (AUC = 0.837), but the testing group exhibited lower accuracy, sensitivity, and specificity (Table 2; Fig. 3).

**Table 2:** The diagnostic performance of different parameters and models for prediction of MACE

Models	Classifiers	AUC(95% CI)	Sensitivity	Specificity	Accuracy	Precision	F1-score
Clinical model (Troponin + CKMB)	LR	0.594 (0.508–0.681) | 0.567 (0.431–0.703)	0.211 | 0.281	0.989 | 0.900	0.63 | 0.625	0.941 | 0.692	0.345 | 0.400
	**RF**	**0.784 (0.714–0.854) | 0.541 (0.406–0.677)**	**0.526 | 1.000**	**0.944 | 0.200**	**0.752 | 0.556**	**0.889 | 0.500**	**0.661 | 0.667**
	SVM	0.595 (0.508–0.681) | 0.567 (0.431–0.703)	0.211 | 0.281	0.989 | 0.900	0.63 | 0.625	0.941 | 0.692	0.345 | 0.400
Imaging model	LR	0.713 (0.634–0.793) | 0.751 (0.634–0.868)	0.539 | 0.906	0.843 | 0.575	0.703 | 0.722	0.745 | 0.630	0.625 | 0.743
	**RF**	**0.784 (0.715–0.853) | 0.785 (0.681–0.890)**	**0.605 | 0.75**	**0.854 | 0.725**	**0.739 | 0.736**	**0.78 | 0.686**	**0.681 | 0.717**
	SVM	0.704 (0.624–0.785) | 0.723 (0.600–0.845)	0.553 | 0.875	0.820 | 0.650	0.697 | 0.750	0.724 | 0.667	0.627 | 0.757
Clinical.Imaging model	LR	0.745 (0.669–0.821) | 0.750 (0.634–0.866)	0.645 | 0.906	0.798 | 0.550	0.727 | 0.708	0.731 | 0.617	0.685 | 0.734
	**RF**	**0.827 (0.765–0.888) | 0.745 (0.632–0.857)**	**0.684 | 0.938**	**0.854 | 0.475**	**0.776 | 0.681**	**0.800 | 0.588**	**0.737 | 0.723**
	SVM	0.723 (0.645–0.802) | 0.726 (0.604–0.848)	0.579 | 0.875	0.798 | 0.625	0.697 | 0.736	0.71 | 0.651	0.638 | 0.747
LADR model	LR	0.717 (0.639–0.794) | 0.645 (0.516–0.773)	0.895 | 0.625	0.472 | 0.65	0.667 | 0.639	0.591 | 0.588	0.712 | 0.606
	**RF**	**0.801 (0.735–0.867) | 0.685 (0.560–0.810)**	**0.842 | 0.531**	**0.674 | 0.825**	**0.752 | 0.694**	**0.688 | 0.708**	**0.757 | 0.607**
	SVM	0.712 (0.634–0.790) | 0.635 (0.505–0.765)	0.908 | 0.656	0.449 | 0.650	0.661 | 0.653	0.585 | 0.600	0.712 | 0.627
LCXR model	LR	0.750 (0.677–0.824) | 0.708 (0.586–0.829)	0.776 | 0.688	0.629 | 0.675	0.697 | 0.681	0.641 | 0.629	0.702 | 0.657
	**RF**	**0.837 (0.778–0.897) | 0.667 (0.540–0.794)**	**0.895 | 0.375**	**0.640 | 0.900**	**0.758 | 0.667**	**0.680 | 0.750**	**0.773 | 0.500**
	SVM	0.726 (0.649–0.803) | 0.726 (0.609–0.843)	0.566 | 0.656	0.820 | 0.700	0.703 | 0.681	0.729 | 0.636	0.637 | 0.646
RCAR model	LR	0.704 (0.625–0.784) | 0.648 (0.519–0.776)	0.671 | 0.812	0.708 | 0.450	0.691 | 0.611	0.662 | 0.542	0.666 | 0.650
	**RF**	**0.799 (0.731–0.866) | 0.626 (0.494–0.758)**	**0.737 | 0.375**	**0.764 | 0.850**	**0.752 | 0.639**	**0.727 | 0.667**	**0.732| 0.480**
	SVM	0.718 (0.64–0.796) | 0.652 (0.525–0.78)	0.789 | 0.625	0.584 | 0.675	0.679 | 0.653	0.619 | 0.606	0.694| 0.615
Radiomics model	LR	0.740 (0.665–0.815) | 0.727 (0.605–0.848)	0.553 | 0.750	0.843 | 0.700	0.709 | 0.722	0.750 | 0.667	0.637 | 0.706
	**RF**	**0.832 (0.772–0.892) | 0.710 (0.588–0.832)**	**0.658 | 0.594**	**0.854 | 0.825**	**0.764 | 0.722**	**0.794 | 0.731**	**0.720 | 0.655**
	SVM	0.739 (0.663–0.815) | 0.746 (0.627–0.865)	0.592 | 0.812	0.843 | 0.700	0.727 | 0.750	0.763 | 0.684	0.667 | 0.743
Radiomics.Clinical model	LR	0.764 (0.691–0.836) | 0.756 (0.641–0.871)	0.592 | 0.719	0.843 | 0.775	0.727 | 0.750	0.763 | 0.719	0.667 | 0.719
	**RF**	**0.856 (0.801–0.912) | 0.713 (0.590–0.835)**	**0.697 | 0.531**	**0.865 | 0.850**	**0.788 | 0.708**	**0.815 | 0.739**	**0.751 | 0.618**
	SVM	0.756 (0.682–0.829) | 0.763 (0.648–0.878)	0.592 | 0.750	0.843 | 0.775	0.727 | 0.764	0.763 | 0.727	0.667 | 0.738
Radiomics.Imaging model	LR	0.786 (0.717–0.856) | 0.798 (0.696–0.899)	0.658 | 0.906	0.798 | 0.575	0.733 | 0.722	0.735 | 0.630	0.694 | 0.743
	**RF**	**0.872 (0.820–0.924) | 0.815 (0.715–0.914)**	**0.776 | 0.906**	**0.820 | 0.625**	**0.800 | 0.750**	**0.787 | 0.659**	**0.781 | 0.763**
	SVM	0.760 (0.687–0.834) | 0.780 (0.674–0.885)	0.697 | 0.938	0.764 | 0.550	0.733 | 0.722	0.716 | 0.625	0.706 | 0.750
Combination model (Radiomics.Clinical.Imaging)	LR	0.809 (0.743–0.874) | 0.805 (0.705–0.904)	0.816 | 0.969	0.652 | 0.525	0.727 | 0.722	0.667 | 0.620	0.734 | 0.756
	**RF**	**0.886 (0.838–0.934) | 0.786 (0.680–0.892)**	**0.763 | 0.688**	**0.876 | 0.825**	**0.824 | 0.764**	**0.841 | 0.759**	**0.800 | 0.722**
	SVM	0.772 (0.701–0.844) | 0.787 (0.683–0.892)	0.697 | 0.906	0.764 | 0.600	0.733 | 0.736	0.716 | 0.644	0.706 | 0.753

Bold values indicate the position of important results

Training cohort | Testing cohort

MACE, major adverse cardiovascular events; AUC, Area Under The Curve; CI, confidence interval; CKMB, creatine kinase MB; LR, Logistic Regression; RF, Random forest; SVM, Support vector machine; LAD, left anterior descending branch radiomics; LCX, left circumflex radiomics; RCA, right coronary artery radiomics

### Performance of combination models

The radiomics model included 10 features, all of which were significantly correlated with MACE (*p* < 0.05) (Fig. S1). Significant differences in these 10 features were found between MACE+ and MACE− groups (*p* < 0.05), except for the LCX_glcm_Imc2 (Fig. S2). The AUC of the Clinical.Imaging model in the training cohort improved compared to the Imaging model (0.827 vs. 0.784, *p* = 0.004). However, the sensitivity of the Clinical.Imaging model was poor at 0.684. The Radiomics.Imaging model demonstrated a higher AUC than the Radiomics model in both the training (0.872 vs. 0.832, *p* = 0.049) and testing (0.815 vs. 0.710, *p* = 0.003) cohort. The combination model (Radiomics.Clinical.Imaging) improved the discriminating capability of MACE, with significantly better specificity, accuracy, and precision, despite demonstrating similar predictive efficiency to the Radiomics.Imaging model in both the training (0.886 vs. 0.872) and testing cohorts (0.786 vs. 0.815). Compared to other models, the combination model shows significantly better prediction efficiency in the training cohort than the Clinical.Imaging model (AUC = 0.886 vs. 0.827, *p* = 0.009) and Radiomics model (0.886vs. 0.832, *p* = 0.009) (Table 3; Fig. 3).

**Table 3:** The comparison of models in training and test cohorts

Model1	Model2	Classifiers	*p* train	*p* test
Radiomics	LCXR	LR	0.421	0.388
		RF	0.749	0.136
		SVM	0.302	0.4
Clinical.Radiomics	Clinical.Imaging	LR	0.696	0.933
		RF	0.404	0.633
		SVM	0.507	0.65
Clinical.Radiomics	Radiomics.Imaging	LR	0.502	0.369
		RF	0.456	0.003*
		SVM	0.915	0.785
Radiomics.Imaging	Clinical.Imaging	LR	0.09	0.159
		RF	0.077	0.18
		SVM	0.015*	0.093
Radiomics.Imaging	Radiomics	LR	0.143	0.118
		RF	0.049*	0.003*
		SVM	0.58	0.581
Radiomics.Clinical.Imaging	Radiomics	LR	0.033*	0.132
		RF	0.009*	0.099
		SVM	0.378	0.51
Radiomics.Clinical.Imaging	Clinical.Imaging	LR	**0.001†**	0.258
		RF	0.009*	0.296
		SVM	**0.001†**	0.045*
Radiomics.Clinical.Imaging	Clinical.Radiomics	LR	0.145	0.249
		RF	0.104	0.04*
		SVM	0.665	0.249
Radiomics.Clinical.Imaging	Radiomics.Imaging	LR	0.039*	0.711
		RF	0.122	0.251
		SVM	0.042*	0.52

Bold values highlight the P-values with statistical differences

*, *p* < 0.05; †, *p* < 0.001

LR, Logistic Regression; RF, random forest; SVM, Support vector machine

### Evaluation and verification of the combination prediction models

**Fig. 4:**
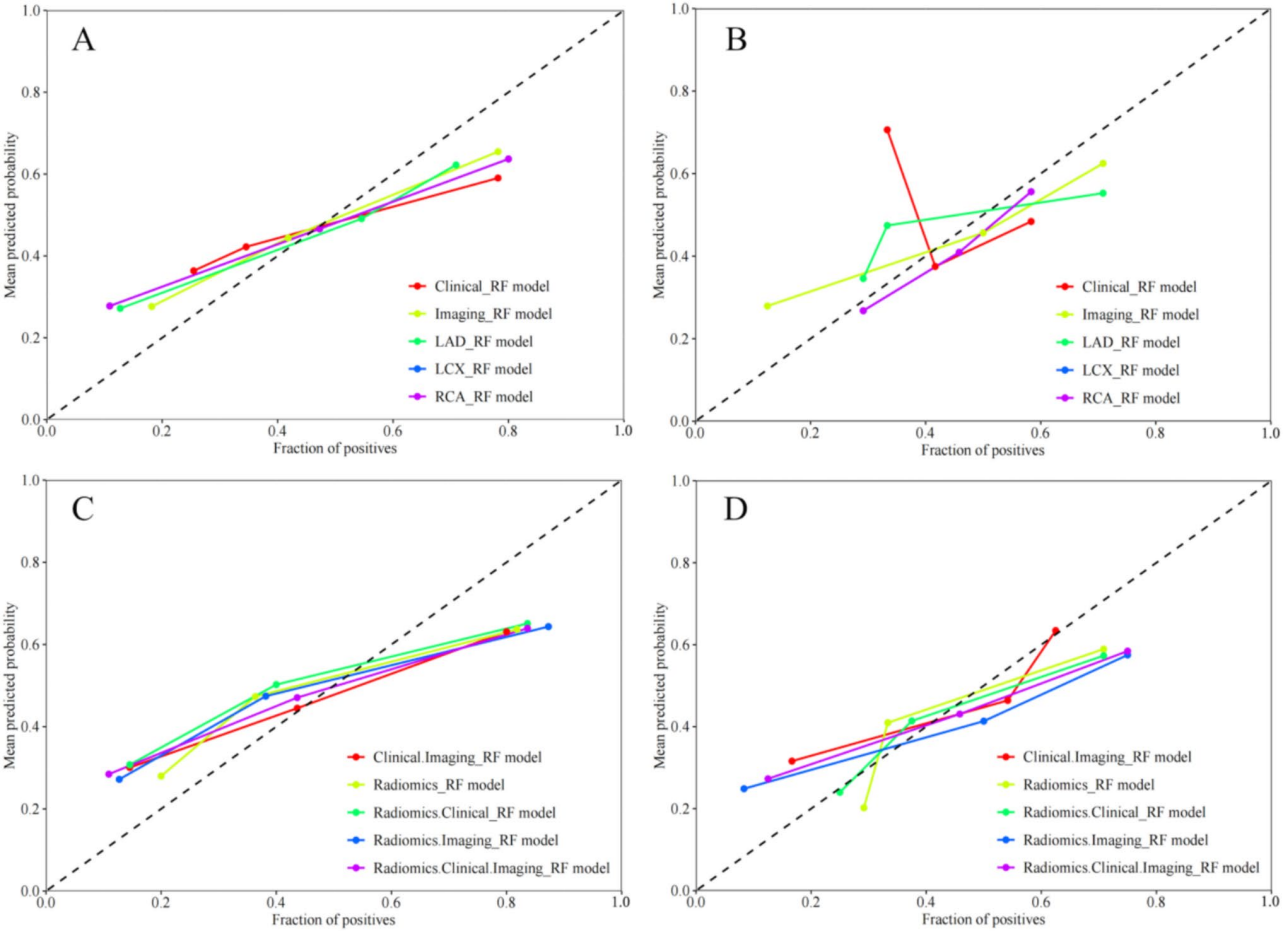
Evaluation and verification of the combination models. Calibration curves of the combination models in terms of agreement between predicted and actual MACE risk in the training (**A**, **C**) and testing (**B**, **D**) cohort. X-axis represents Mace prediction, Y-axis represents actual MACE status, and dashed line represents the ideal prediction

**Fig. 5:**
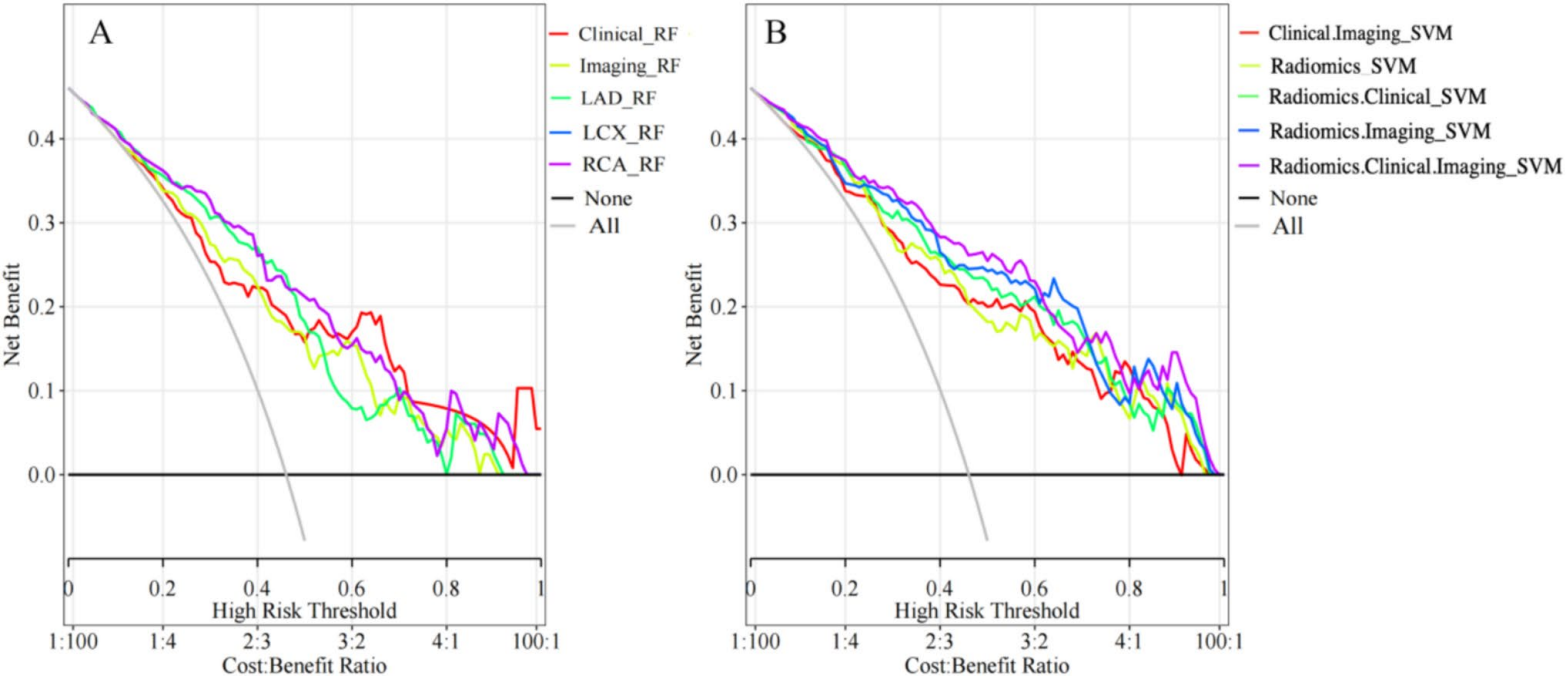
Decision curves of the combination models in training (**A**) and testing (**B**) cohort. The gray line represents the assumption that all patients with MACE, and the horizontal black line represents the assumption that no patient with MACE

Figure 2 shows the construction process of the MACE prediction model in patients with hypertension and CAD within two years. The predictive models’ calibration curves demonstrated a good fit between prediction and observation for the probability of MACE in both training and testing cohorts (Fig. 4). The Hosmer–Lemeshow test confirmed this with all *p*-values of > 0.05. The combination model (Radiomics.Clinical.Imaging) had the Brier score of 0.016 (Table 4). The decision curves demonstrated the clinical applicability of predictive models and the combination model had the highest net benefit over a wider range by comparing the net benefits at different threshold probabilities in the testing cohorts (Fig. 5). Figure S3 shows the heatmap of the correlation between radiomics features and clinicoradiological features used for model development.

**Table 4:** The Hosmer–Lemeshow test of models

Models	*p* value of Hosmer–Lemeshow test			Brier score		
	LR	RF	SVM	LR	RF	SVM
Clinical	0.102	0.239	0.078	0.102	**0.001**	0.093
Imaging	0.143	0.282	0.246	0.009	**< 0.001**	0.012
Clinical.Imaging	0.198	0.406	0.220	0.009	**< 0.001**	0.007
LADR	0.456	0.100	0.206	0.005	**< 0.001**	0.003
LCXR	0.760	0.707	0.636	< 0.001	**0.043**	0.005
RCAR	0.760	0.707	0.636	< 0.001	**0.043**	0.005
Radiomics	0.223	0.464	0.103	0.009	**0.003**	0.003
Radiomics.Clinical	0.482	0.557	0.197	< 0.001	**0.007**	0.009
Radiomics.Imaging	0.403	0.028	0.165	0.007	**0.019**	0.003
Radiomics.Clinical.Imaging	0.276	0.058	0.453	0.007	**0.016**	0.003

LR, Logistic Regression; RF, Random forest; SVM, Support vector machine

## Discussion

In this study, we established a multiparametric model for predicting MACE in hypertension combined CAD patients preoperatively. The combination model that integrates CT-FFR, clinicoradiological features and selected radiomics features of PCAT demonstrated superior diagnostic performance in predicting MACE in patients with hypertension and CAD over a two-year follow-up period.

Hypertension is one of the major risk factors for CAD in its various clinical aspects (myocardial infarction, angina pectoris, acute coronary syndrome, and chronic coronary syndromes). The interplay between hypertension and CAD exacerbates the risk of MACE; the incidence of MACE was significantly higher than with either condition alone [22]. Existing prediction models mainly focus on the risk assessment of a single disease, failing to capture the intricate interactions between hypertension and CAD, thus underestimating their combined risks [23, 24]. By focusing on this high-risk group, our study bridges this gap by developing a MACE prediction model that accurately reflects the complex pathophysiology and provides meaningful insights for clinical practice in patients with hypertension and CAD.

### Prediction of MACE using traditional clinicoradiological methods

The traditional prediction model established in this study had low efficiency in predicting MACE, which was similar to the previous study. Numerous clinical studies have confirmed that myocardial ischemia severity does not always correlate with the degree of coronary anatomical stenosis. This discrepancy reduces the CCTA value in assessing functional myocardial ischemia. Furthermore, the CT-FFR (based on either computational fluid dynamics or machine-learning approaches) is limited for lesions in the “gray zone” with CT-FFR values of 0.7–0.8 [6, 25, 26]. The PCAT emphasizes the potential of complementary imaging biomarkers. Vascular inflammation triggers permanent alterations in the perivascular space, such as fibrosis and neoangiogenesis [27]. The CRISP-CT study investigates the ability of perivascular FAI to predict all-cause and cardiac mortality, revealing its capability to capture dynamic changes in water–lipid balance within inflamed PCAT. However, a subgroup of the CRISP-CT study receiving post-CCTA treatment with statins and aspirin revealed that FAI lost its prognostic value, implying modifiability of the risk identified by FAI. This highlights the necessity for additional biomarkers capable of detecting permanent changes in PCAT composition less influenced by treatments targeting vascular inflammation [21]. Consequently, the association between ischemic stenosis and PCAT may not be adequately represented solely by absolute PCAT attenuation, which fails to provide the spatial distribution of voxel gray-level intensities and quantify heterogeneity.

### Prediction of MACE using radiomics models

Complex mathematical formulas, which are invisible to the naked eye of experienced radiologists and clinicians, are used to transform radiomic approaches to qualitative imaging features into quantitative variables. Radiomics has found extensive use in oncology [28, 29], and its application in cardiovascular imaging, particularly in capturing advanced features of coronary plaque phenotype, remains emerging [30]. Our radiomics model based on PCAT achieved an AUC of 0.832 in the training group and 0.710 in the testing group, demonstrating modest prediction sensitivity. We integrated these radiomics biomarkers with clinicoradiological data, including coronary stenosis, plaque load, and CT-FFR, to construct comprehensive models for determining high-risk MACE patients to improve predictive accuracy. Radiomic parameters added incremental discriminatory value over and above PCAT attenuation and clinical features in this combination models with AUCs of 0.886 and 0.786 in the training and testing datasets, respectively, demonstrating excellent specificity and sensitivity. Furthermore, all 10 extracted omics features in this study demonstrated close associations with MACE, indicating a potential association between PCAT radiomic phenotype and plaque rupture with associated local inflammatory responses. Our model enhances predictive power and provides a comprehensive tool for clinicians, facilitating early intervention and aggressive secondary prevention strategies for individuals at high risk of MACE and potentially improving clinical outcomes. The decision and clinical impact curves further affirm the model’s utility, highlighting its relevance in guiding treatment strategies and optimizing resource use in clinical settings.

There are several limitations in our study. We did not evaluate PCAT at the per-lesion level due to the time-consuming nature, human factor susceptibility, and low plaque delineation reproducibility. Histopathologic and invasive imaging studies revealed diffuse coronary inflammation in patients with acute myocardial infarction [31]; atherosclerosis-induced PCAT changes may exist beyond the length of the plaque [32]. Instead, we investigated adipose tissue at the per-vessel level, which significantly improved study reproducibility and efficiency. Lesions with severe calcification were not excluded; they potentially affect CT-FFR accuracy. However, risk assessment for these patients should not be overlooked, which is also of great clinical concern and is the value of the combination model. Moreover, subgroup analysis to identify the incremental value of the PCAT radiomics model in “gray-zone lesions” was not feasible due to the limited sample size; subgroup analysis will be conducted in future studies to address this gap. Finally and importantly, larger cohorts from other centers need to be enrolled for the prospective validation of this MACE prediction model in larger and more diverse populations.

In summary, the combination prediction model, which incorporates clinicoradiological features and radiomics features of PCAT, has important supplementary value for predicting MACE in people with hypertension and CAD.

## Supplementary Information

Below is the link to the electronic supplementary material.

Supplementary file1 (PDF 705 kb)
