# Supplementary material for: Multimodal prediction of major adverse cardiovascular events in hypertensive patients with coronary artery disease: integrating pericoronary fat radiomics, CT-FFR, and clinicoradiological features
Source: Radiol Med. 2025 Mar 21;130(6):767–81. doi: 10.1007/s11547-025-01991-3 (PMC12185633; doi:10.1007/s11547-025-01991-3)
Supplement: Supplementary file 1 — Supplementary file1 (PDF 705 kb) [file 11547_2025_1991_MOESM1_ESM.pdf]

Supplemental Information

Multimodal Prediction of Major Adverse Cardiovascular Events in Hypertensive Patients with Coronary Artery Disease: Integrating Pericoronary Fat Radiomics, CT-FFR, and Clinicoradiological Features

La radiologia medica

Qing Zou, MD[1*], Taichun Qiu, PD[1], Chunxiao Liang, PD[1], Fang Wang, MD[2], Yongji Zheng, PD[1], Jie Li, PD[1], Xingchen Li, PD[1], Yudan Li, PD[1], Zhongyan Lu, PD[1], Dongmei Xie, PD[1], Qingtao Hui, BD[1], Hongyi Deng, BD[1], Bing Ming, PD[1*]

[1] Department of Radiology, Deyang People's Hospital, 173# Section 3 Tai Shan Road, Deyang, Sichuan, China, 618400.

[2] Department of Research and Development, Shanghai United Imaging Intelligence Co., Ltd. Shanghai, China, 200232.

Guarantor and correspondent:

Qing Zou, MD

Department of Radiology, Deyang People's Hospital, 173# Section 3 Tai Shan Road, Deyang, Sichuan 618400, China.

Tel: +86 15283804266

E-mail: yezideshu@163.com

Table S1

Filters	first-order	shape	glcm	gldm	glrlm	glszm	ngtdm	sum
original	18	14	21	14	16	16	5	104
additivegaussiannoise	18		21	14	16	16	5	90
binomialblurimage	18		21	14	16	16	5	90
boxmean	18		21	14	16	16	5	90
boxsigmaimage	18		21	14	16	16	5	90
curvatureflow	18		21	14	16	16	5	90
discretegaussian	18		21	14	16	16	5	90
laplaciansharpening	18		21	14	16	16	5	90
mean	18		21	14	16	16	5	90
normalize	18		21	14	16	16	5	90
recursivegaussian	18		21	14	16	16	5	90
shotnoise	18		21	14	16	16	5	90
specklenoise	18		21	14	16	16	5	90
wavelet	144		168	112	128	128	40	720
sum	378	14	441	294	336	336	105	1904

GLCM: Gray Level Co-occurrence Matrix, GLDM: Gray Level Dependence Matrix, GLSZM: Gray Level Size Zone Matrix, GLRLM: Gray Level Run Length Matrix, NGTDM: Neighbouring Gray Tone Difference Matrix

Table S2 Logistics regression analysis of factors associated with MACE

Clinical Factor	Univariate		Multivariate	
	P value	OR（95% CI）	P value	OR（95% CI）
**Demographic characteristics**				
Age	0.718	1.006(0.974-1.039)		
Gender	0.995	1.002(0.533-1.887)		
Height	0.740	0.993(0.951-1.036)		
Weight	0.778	1.004(0.974-1.036)		
SBP	0.943	1.000(0.988-1.012)		
DBP	0.590	0.994(0.974-1.015)		
Hypertension history	0.411	1.016(0.978-1.057)		
NYHA grade	0.050	1.382(1.004-1.922)	0.206	1.263(0.878-1.818)
COPD	0.812	1.105(0.479-2.535)		
Hyperlipidemia	0.276	1.531(0.712-3.333)		
Chronic kidney diseases	0.081	2.286(0.920-6.045)	0.634	1.288(0.453-3.739)
Diabetes	**0.042**	1.906(1.028-3.568)	0.204	1.818(0.722-4.606)
Antihypertense treatment	0.364	1.448(0.658-3.289)		
Statin use	0.088	1.716(0.926-3.210)	0.062	1.931(0.975-3.904)
Smoking	0.342	1.406(0.696-2.854)		
Drinking	0.280	1.559(0.697-3.542)		
DLP	0.592	1.001(0.998-1.003)		
**Laboratory characteristics**				
FT3	**0.032**	0.576(0.341-0.914)		
FT4	0.454	1.044(0.934-1.173)		
TSH	0.101	0.866(0.722-1.019)		

TGAb	0.636	1.000(0.999-1.002)		
TPOAb	0.809	0.999(0.994-1.004)		
C-reactive protein	0.201	1.010(0.995-1.026)		
White blood cell count	0.450	1.051(0.925-1.199)		
Creatinine	0.535	1.001(0.998-1.004)		
Troponin	0.084	1.333(1.063-2.020)	0.150	1.269(1.013-1.958)
CKMB	**0.026**	1.156(1.037-1.345)	0.173	1.102(0.972-1.298)
MYO	0.211	1.002(0.999-1.006)		
**Imaging characteristics**				
LAD DS	**0.008**	1.383(1.094-1.769)	0.428	0.781(0.418-1.442)
LCX DS	**< 0.01**	1.605(1.280-2.042)	0.096	1.368(0.950-1.996)
RCA DS	**0.003**	1.403(1.124-1.768)	0.462	0.857(0.565-1.290)
DS patients	**0.001**	1.573(1.208-2.087)	0.330	1.434(0.697-3.009)
Calcification score	0.293	1.000(1.000-1.000)		
Involved segments	**0.005**	1.147(1.044-1.266)	0.638	0.963(0.818-1.128)
LAD CT-FFR	**< 0.01**	0.014(0.001-0.130)	0.089	0.079(0.003-0.875)
LCX CT-FFR	**0.004**	0.028(0.002-0.282)	0.797	0.649(0.022-17.207)
RCA CT-FFR	**< 0.01**	0.010(0.001-0.104)	0.119	0.063(0.002-1.834)
LAD FAI	0.099	1.028(0.995-1.064)	0.842	0.993(0.928-1.063)
LCX FAI	**0.023**	1.044(1.007-1.084)	0.532	1.027(0.944-1.118)
RCA FAI	**0.048**	1.028(1.001-1.057)	0.989	1.000(0.949-1.054)
LAD Fat volume	0.541	1.000(0.999-1.000)		
LCX Fat volume	**0.024**	0.999(0.999-1.000)	0.677	1.000(0.999-1.001)
RCA Fat volume	0.091	1.000(0.999-1.000)	0.838	1.000(0.999-1.001)
Positive remodeling	0.755	1.103(0.596-2.041)		

Punctate calcification	0.081	2.286(0.920-6.045)	0.464	1.555(0.481-5.233)
Napkin Ring	0.561	1.440(0.417-5.186)		
Low density spot	0.328	1.406(0.710-2.796)		
HRP	**0.044**	2.013(1.023-4.021)	0.497	0.708(0.253-1.895)

MACE, major adverse cardiovascular events; OR, odds ratio; CI, confdence interval; SBP, systolic blood pressure; DBP, diastolic blood pressure; NYHA, new york heart association; COPD, chronic obstructive pulmonary disease; DLP, dose-length product; FT3, free triiodothyronine; FT4, free thyroxine; TSH, thyroid-stimulating hormone; TGAb, thyroglobulin antibody; TPOAb, thyroid peroxidase antibody; CKMB, creatine kinase MB; MYO, myoglobin; DS, diameter CT-FFR computed tomography-based fractional fow reserve; FAI, perivascular fat attenuation index.; HRP, high-risk plaques

Fig S1

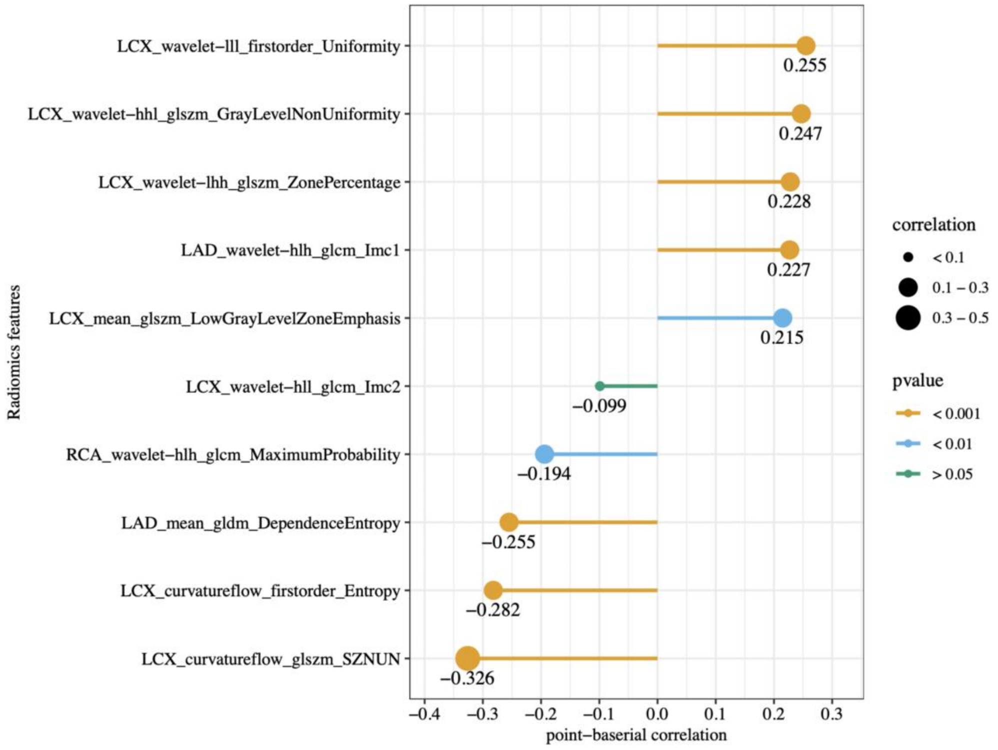

Fig S1 Correlation analysis between Radiomics features and MACE. Only the LCX_wavelet-hlh_glcm_Imc2 (green bar) feature was not significantly correlated with MACE, with a correlation coefficient of less than 0.1 and a correlation test p-value greater than 0.05

Fig S2

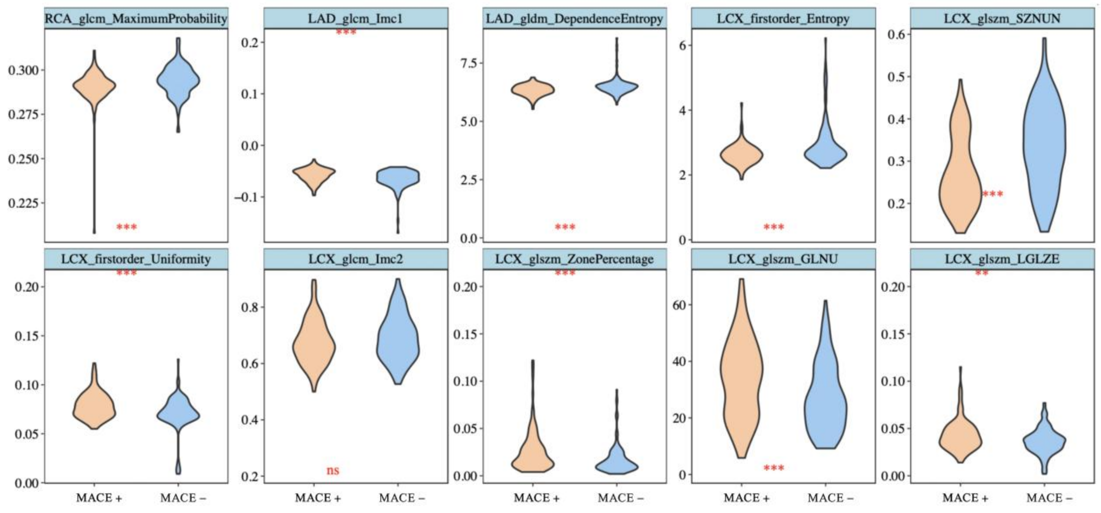

Fig S2 Violin plot of Radiomics features between MACE+ and MACE- group. ****, p<0.001; ***, p<0.01, **, p<0.05; *, p<0.1; ns, p>0.1

Fig S3

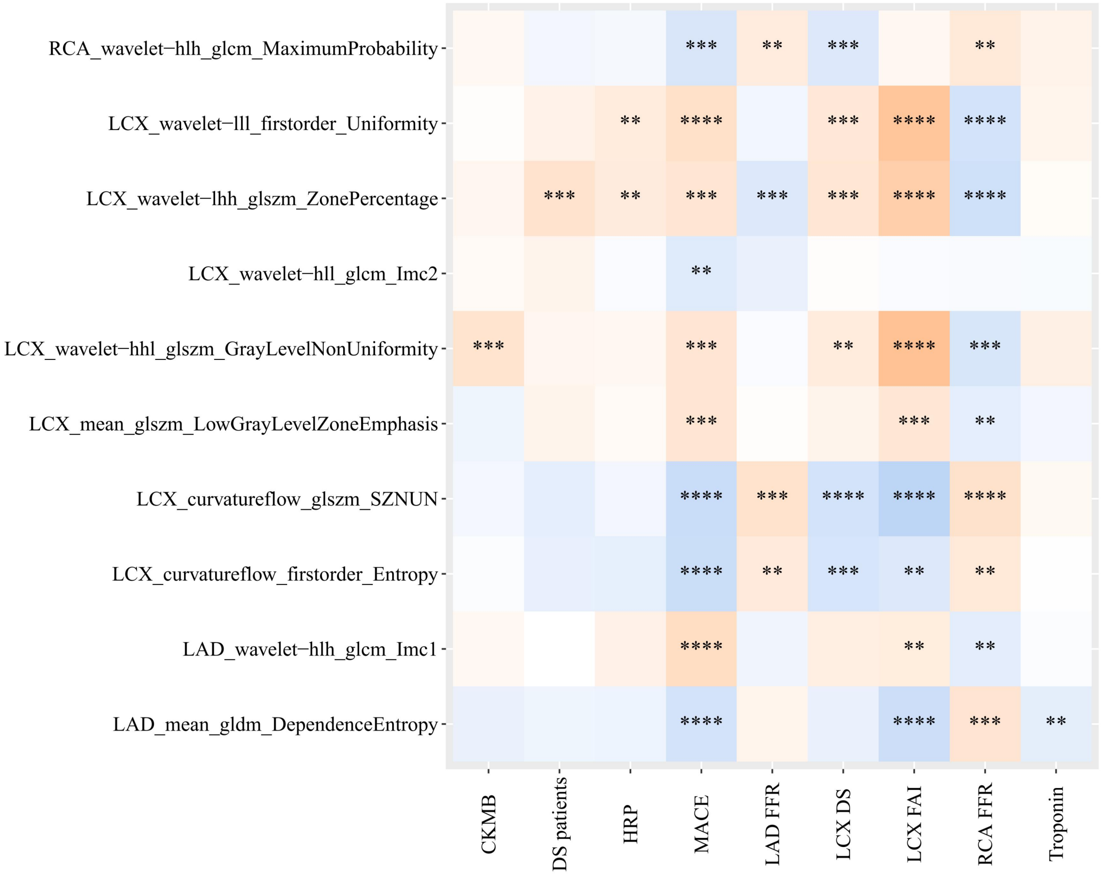

Fig S3 The heatmap of the correlation between MACE, clinicoradiological and radiomics features. ****, p<0.001; ***, p<0.01; **, p<0.05; *,p<0.1
